# Evaluation of a Novel Commercial Real-Time PCR Assay for the Simultaneous Detection of *Cryptosporidium* spp., Giardia duodenalis, and Entamoeba histolytica

**DOI:** 10.1128/spectrum.00531-22

**Published:** 2022-05-03

**Authors:** Alejandro Dashti, Henar Alonso, Cristina Escolar-Miñana, Pamela C. Köster, Begoña Bailo, David Carmena, David González-Barrio

**Affiliations:** a Parasitology Reference and Research Laboratory, National Centre for Microbiology, Carlos III Health Institute, Madrid, Spain; b Department of Microbiology, Paediatrics, Radiology, and Public Health, Faculty of Medicine, University of Zaragoza, Saragossa, Spain; c Department of Animal Production and Food Science, Faculty of Veterinary, University of Zaragoza, Saragossa, Spain; d Centro de Investigación Biomédica en Red de Enfermedades Infecciosas, Instituto de Salud Carlos III, Madrid, Spain; University of Arizona/Banner Health

**Keywords:** molecular diagnostics, multiplex real-time PCR, gastrointestinal parasites, evaluation, *Cryptosporidium*, *Entamoeba histolytica*, *Giardia*

## Abstract

*Cryptosporidium* spp., Giardia duodenalis, and Entamoeba histolytica are the most common diarrhea-causing protozoan species globally. Misdiagnosis is a concern for asymptomatic and chronic infections. Multiplexing, i.e., the detection of more than one parasite in a single test by real-time PCR, allows high diagnostic performance with favorable cost-effectiveness. We conducted a clinical evaluation of the VIASURE *Cryptosporidium*, Giardia, & E. histolytica real-time PCR assay (CerTest Biotec, San Mateo de Gállego, Spain) against a large panel (*n* = 358) of well-characterized DNA samples positive for *Cryptosporidium* spp. (*n* = 96), G. duodenalis (*n* = 115), E. histolytica (*n* = 25), and other parasitic species of the phyla Amoebozoa (*n* = 11), Apicomplexa (*n* = 14), Euglenozoa (*n* = 8), Heterokonta (*n* = 42), Metamonada (*n* = 37), Microsporidia (*n* = 4), and Nematoda (*n* = 6). DNA samples were obtained from clinical stool specimens or cultured isolates in a national reference center. Estimated sensitivity and specificity were 0.96 and 0.99 for *Cryptosporidium* spp., 0.94 and 1 for G. duodenalis, and 0.96 and 1 for E. histolytica, respectively. Positive and negative predictive values were calculated as 1 and 0.98 for *Cryptosporidium* spp., 0.99 and 0.98 for G. duodenalis, and 1 and 0.99 for E. histolytica, respectively. The assay identified six *Cryptosporidium* species (Cryptosporidium hominis, Cryptosporidium parvum, Cryptosporidium canis, Cryptosporidium felis, Cryptosporidium scrofarum, and Cryptosporidium ryanae) and four G. duodenalis assemblages (A, B, C, and F). The VIASURE assay provides rapid and accurate simultaneous detection and identification of the most commonly occurring species and genetic variants of diarrhea-causing parasitic protozoa in humans.

**IMPORTANCE** Thorough independent assessment of the diagnostic performance of novel diagnostic assays is essential to ascertain their true usefulness and applicability in routine clinical practice. This is particularly true for commercially available kits based on multiplex real-time PCR aimed to detect and differentiate multiple pathogens in a single biological sample. In this study, we conducted a clinical evaluation of the VIASURE *Cryptosporidium*, Giardia, & E. histolytica real-time PCR assay (CerTest Biotec) for the detection and identification of the diarrhea-causing enteric protozoan parasites *Cryptosporidium* spp., G. duodenalis, and E. histolytica. A large panel of well-characterized DNA samples from clinical stool specimens or cultured isolates from a reference center was used for this purpose. The VIASURE assay demonstrated good performance for the routine testing of these pathogens in clinical microbiological laboratories.

## INTRODUCTION

Intestinal protozoa continue to be the most commonly encountered parasitic diseases, affecting millions of people each year and causing significant morbidity and deaths worldwide (1). As an example, *Cryptosporidium* infection is the second major cause of moderate to severe diarrhea in children younger than 2 years of age in low-income countries (2). These pathogens are also a public health concern in medium- to high-income countries (3). Indeed, *Cryptosporidium* spp., Giardia duodenalis, and Entamoeba histolytica account for up to 70% of the gastrointestinal parasites diagnosed every year at hospital-based microbiology laboratories in Europe (4, 5). Additionally, both *Cryptosporidium* spp. and G. duodenalis have been increasingly recognized as causative agents of waterborne and foodborne gastrointestinal disease outbreaks in several European countries (6–9).

Traditional diagnostic methods for the detection of intestinal protozoa are based on microscopic examination of fecal material (10). The simplicity and low cost of this method make it suited for clinical laboratories with limited resources, especially in areas with endemicity and high prevalence rates. However, microscopy is labor-intensive and time-consuming, requires highly trained personnel, and is hampered by subjectivity and low sensitivity (11, 12). These features make microscopy less adequate for routine diagnosis in high-income countries, where parasite prevalence rates and burdens are often low (13). Additionally, only moderate agreement in detection rates and thus diagnosis of intestinal protozoa using microscopy was achieved among European reference laboratories (14). In this epidemiological and clinical scenario, highly sensitive PCR-based methods clearly outperform microscopy in the detection of the chosen targets (15, 16). Furthermore, PCR testing of a single stool sample is still more sensitive than the sequential sampling required for microscopic detection (17). Other benefits of molecular diagnostics include (i) reduced hands-on and turnaround times, (ii) high-throughput stool screening, (iii) the possibility of automation, (iv) quantification of the pathogen load as a potential indicator of disease severity, and (v) tailored and cost-efficient implementation in routine diagnostic algorithms of clinical laboratories according to their specific needs (15, 16, 18–20).

The application of multiplex real-time quantitative PCR (qPCR) in molecular diagnostics has boosted the willingness of well-equipped laboratories in western countries, mainly in Europe, to radically adapt their diagnostic algorithms and introduce high-throughput DNA-detecting assays (16). In these clinical settings, molecular diagnostic approaches are inexorably replacing conventional microscopy as first-line routine diagnostic methods for intestinal protozoan parasites (21). Consequently, a wide diversity of commercial multiplex qPCR assays have been developed for this purpose (Table 1).

**TABLE 1:** Diagnostic performance of commercially available real-time PCR assays for the simultaneous detection of *Cryptosporidium* spp., Giardia duodenalis, and Entamoeba histolytica

Assay	Manufacturer	Automated DNA extraction	Pathogen species	PPA (%)^*a*^	NPA (%)^*b*^	PPV (%)^*c*^	NPV (%)	Comparator (reference) method
AllPlex	Seegene	Yes	*Cryptosporidium* spp.	78	92	78	93	PCR (29)
			G. duodenalis	91	95	98	95	
			E. histolytica	100	100	100	100	
BD MAX	BD	Yes	*Cryptosporidium* spp.	96–100	100	92–95	99–100	MC, multiplex qPCR, SS (22, 23); DFA (23)
			G. duodenalis	97–98	98–99	93–97	98–99.9	
			E. histolytica	92–100	100	^*d*^	^*d*^	
EasyScreen	Genetic Signatures	Optional	*Cryptosporidium* spp.	100	100	55–89	92–98	MC, qPCR (24)
			G. duodenalis	92	100	73–96	88–98	
			E. histolytica	92	100	75–100	89–100	
FilmArray	BioFire	Yes	*Cryptosporidium* spp.	100	100	100	99.6	PCR, SS (25)
			G. duodenalis	83–100	99	100	99.5	
			E. histolytica	100	100	NS	100	
FTD stool parasites	Fast Track	No	*Cryptosporidium* spp.	53–64	100	100	93	PCR (29, 30); MC (26)
			G. duodenalis	90–100	93	74	98	
			E. histolytica	100	100	100	100	
Gastroenteritis/parasite panel I	Diagenode	No	*Cryptosporidium* spp.	74–75	99	96–96	95–100	MC, ELISA (4); PCR (29, 30)
			G. duodenalis	68–76	97	87–92	94–98	
			E. histolytica	100	100	80–100	99–100	
NanoCHIP	Savyon Diagnostics	No	*Cryptosporidium* spp.	98–100	95–100	100	100	MC (27, 28), XC, ELISA (28)
			G. duodenalis	98–100	95–100	82	100	
			E. histolytica	98–100	95–100	100	100	
RIDAGENE	R-Biopharm	No	*Cryptosporidium* spp.	87	100	94	96	PCR (29)
			G. duodenalis	79	98	96	88	
			E. histolytica	67	100	100	100	
VIASURE	CerTest Biotec	No	*Cryptosporidium* spp.	100	99	97	100	PCR (30)
			G. duodenalis	81	99	93	96	
			E. histolytica	100	100	100	100	

a Positive percent agreement (PPA) values are calculated identically to sensitivity values. In this study, positive percent agreement is used in place of sensitivity to acknowledge the comparator´s uncertainty.

b Negative percent agreement (NPA) values are calculated identically to specificity. In this study, negative percent agreement is used in place of specificity to acknowledge the comparator´s uncertainty.

c PPV, positive predictive value; NPV, negative predictive value; NS, not specified; DFA, direct fluorescent-antibody assay; ELISA, enzyme-linked immunosorbent assay; MC, microscopy; SS, Sanger sequencing, XC, xenic culture.

d Percent agreement was not calculated for this parasite, due to the inability of microscopy to differentiate between E. histolytica and E. dispar.

Validation and standardization of novel diagnostic assays and procedures are some of the main tasks conducted by national reference centers, because these institutions are able to bring together the resources (e.g., biological samples for reference and equipment) and the expertise to perform the tasks efficiently. Here, we aimed to evaluate the clinical diagnostic performance of the VIASURE *Cryptosporidium*, Giardia, & E. histolytica real-time PCR assay (CerTest Biotec, San Mateo de Gállego, Spain) for the simultaneous detection and differentiation of *Cryptosporidium* spp., Giardia duodenalis, and Entamoeba histolytica.

(The preliminary results of this study were presented at the 31st European Congress of Clinical Microbiology and Infectious Diseases, 9 to 12 July 2021.)

## RESULTS

The VIASURE assay correctly identified 94.8% (91/96 samples) of the DNA samples that were positive for *Cryptosporidium* spp. (Tables 2 and 3). The assay recognized isolates belonging to six distinct *Cryptosporidium* species, including primarily anthroponotic Cryptosporidium hominis (*gp60* subtype families Ia, Ib, and Ie), zoonotic Cryptosporidium parvum (*gp60* subtype families IIa, IIc, and IId), canine-adapted Cryptosporidium canis, feline-adapted Cryptosporidium felis, bovine-adapted Cryptosporidium ryanae, and swine-adapted Cryptosporidium scrofarum (see Table S1 in the supplemental material). No cross-reactions were observed with DNA samples positive for other microeukaryotic (including apicomplexa of the genera *Babesia*, *Besnoitia*, *Isospora*, *Neospora*, *Plasmodium*, *Sarcocystis*, or *Toxoplasma*) and nematode parasites (see Table S1). Of note, 18 *Cryptosporidium*-positive samples were concomitantly infected by G. duodenalis, as previously determined during routine initial diagnosis. All 18 Giardia infections were also detected by the VIASURE assay (see Table S1).

**TABLE 2:** Panel of laboratory-confirmed DNA samples used for diagnostic evaluation of the CerTest VIASURE gastrointestinal panel II real-time PCR assay in the present study

Phylum	Genus	Species	No. of DNA isolates
Apicomplexa	*Cryptosporidium*	C. hominis	73
		C. parvum	17
		C. canis	1
		C. felis	2
		C. ryanae	1
		C. scrofarum	2
Metamonada	Giardia	G. duodenalis	115
Amoebozoa	*Entamoeba*	E. histolytica	25
		*E. dispar*	11
Apicomplexa	*Babesia*	*B. divergens*	1
	*Besnoitia*	*B. besnoiti*	2
	*Cystoisospora*	*C. belli*	1
	*Neospora*	N. caninum	1
	*Plasmodium*	P. falciparum	1
		*P. malariae*	1
		*P. ovale*	1
		P. vivax	1
	*Sarcocystis*	*S. arctica*	1
		*S. cruzi*	1
		*S. gigantea*	1
	*Toxoplasma*	T. gondii	2
Euglenozoa	*Leishmania*	*L. aethiopica*	1
		L. amazonensis	1
		*L. braziliensis*	1
		L. donovani	1
		L. infantum	1
		L. major	1
		L. mexicana	1
		*L. tropica*	1
Heterokonta	*Blastocystis*	*Blastocystis sp.*	42
Metamonada	*Dientamoeba*	*D. fragilis*	37
Microsporidia	*Enterocytozoon*	E. bieneusi	4
Nematoda	*Anisakis*	*A. simplex*	1
	*Dirofilaria*	*D. repens*	1
	*Loa*	*L. loa*	1
	*Mansonella*	*M. perstans*	1
	*Oncocerca*	*O. volvulus*	1
	*Trichuris*	*T. muris*	1
Total			358

**TABLE 3:** Direct comparison of the CerTest VIASURE *Cryptosporidium*, Giardia, & E. histolytica real-time PCR assay with reference PCR methods used during routine analyses at initial diagnosis

Protozoan species	No. with VIASURE assay/reference assay results of:				Kappa
	Positive/positive	Positive/negative	Negative/positive	Negative/negative	
*Cryptosporidium* spp.	91	0	5	262	0.964
Giardia duodenalis	111	1	4	242	0.968
Entamoeba histolytica	24	0	1	333	0.978

Regarding G. duodenalis, the VIASURE assay accurately detected 96.5% (111/115 samples) of the DNA samples that were positive for this protozoon, including zoonotic assemblages A and B, canine-adapted assemblage C, and feline-adapted assemblage F (Table 3; also see Table S1). No cross-reactions were observed with DNA samples positive for other intestinal parasites, including closely related members of the phylum Metamonada, such as Dientamoeba fragilis (see Table S1). The VIASURE assay also detected 5 *Cryptosporidium* coinfections (3 C. hominis coinfections, 1 C. canis coinfection, and 1 C. felis coinfection) that were previously identified during routine initial diagnosis.

Similarly, the VIASURE assay correctly identified 96.0% (24/25 samples) of the DNA samples that were positive for E. histolytica without cross-reactions with other enteric parasites, including closely related members of the phylum Amoebozoa such as Entamoeba dispar (Table 3; also see Table S1).

The VIASURE assay reported 1 potential false-positive result for G. duodenalis (belonging to the cross-reactivity panel) and 10 potential false-negative results (5 samples with *Cryptosporidium* spp., 4 samples with G. duodenalis, and 1 sample with E. histolytica). Reassessment of the 5 samples with *Cryptosporidium* spp. (4 samples with C. hominis and 1 sample with C. parvum) using the singleplex PCR assay used at initial diagnosis as the reference method yielded positive results in all 5 cases, confirming the VIASURE assay results as false-negative results. Reassessment of the 4 G. duodenalis samples and the single E. histolytica sample using the corresponding singleplex PCR assays used at initial diagnosis as the reference method yielded positive results (range of cycle threshold [*C_T_*] values, 30.9 to 41.0) in all cases, confirming the VIASURE assay results as false-negative results.

None of the 122 DNA samples that were positive for parasite species other than *Cryptosporidium* spp., G. duodenalis, and E. histolytica generated false-positive results for these three pathogens as a consequence of undesired cross-reactions. However, 13 of the samples harbored coinfections with G. duodenalis, 1 with *Cryptosporidium* spp., and 1 with G. duodenalis plus C. hominis, all of which were previously detected at initial diagnosis (see Table S1). Overall, very good agreement (kappa test values of ≥0.96) was observed between the results obtained by the VIASURE assay and those previously generated by the reference singleplex PCR assays at initial diagnosis (Table 3).

Taking singleplex PCR results obtained during routine initial diagnosis as the reference, the diagnostic performance of the VIASURE assay is summarized in Table 4. In brief, sensitivity values for all three protozoan parasites ranged from 0.94 to 0.96, specificity values from 0.99 to 1, positive predictive values from 0.99 to 1, and negative predictive values from 0.98 to 0.99.

**TABLE 4:** Diagnostic performance of the VIASURE *Cryptosporidium*, Giardia, & E. histolytica real-time PCR detection assay using as references samples confirmed by PCR during routine analyses at initial diagnosis

Protozoan species	Overall agreement	No.^*a*^				Sensitivity	Specificity	PPV	NPV
		TP	TN	FP	FN				
*Cryptosporidium* spp.	0.98 (0.96–0.99)	91	262	0	5	0.96 (0.91–0.99)	0.99 (0.97–1)	1 (0.94–1)	0.98 (0.95–0.99)
Giardia duodenalis	0.98 (0.96–0.99)	111	242	1^*b*^	4	0.94 (0.88–0.98)	1 (0.98–1)	0.99 (0.94–0.99)	0.98 (0.95–0.99)
Entamoeba histolytica	0.99 (0.98–1)	24	333	0	1	0.96 (0.79–0.99)	1 (0.98–1)	1 (0.82–1)	0.99 (0.98–0.99)
All three	0.96 (0.94–0.98)	226	121	1	10	0.95 (0.92–0.97)	0.99 (0.99–1)	0.99 (0.97–0.99)	0.92 (0.86–0.96)

a TP, true positive; TN, true negative; FP, false positive; FN, false negative; PPV, positive predictive value; NPV, negative predictive value.

b Sample reconfirmed by individual qPCR.

## DISCUSSION

We carried out a comprehensive evaluation of the diagnostic performance of the CerTest VIASURE *Cryptosporidium*, Giardia, & E. histolytica real-time PCR assay for the detection and identification of the three most clinically relevant intestinal protozoan parasites, namely, *Cryptosporidium* spp., Giardia duodenalis, and Entamoeba histolytica. A major methodological contribution is the use of a large reference panel of well-characterized DNA samples. Most evaluation studies conducted previously were based on prospectively collected stool samples submitted to clinical laboratories for parasite investigation (4, 12, 22–28), whereas studies based on selected DNA panels were far less common (29, 30). The latter approach allows additional benefits, including the inclusion of DNA samples from less prevalent or rare species/genotypes and animal-adapted genetic variants with zoonotic potential. This is an important issue because, for instance, qPCR performances for *Cryptosporidium* species other than C. hominis and C. parvum, which can account for nearly 10% of human cases of cryptosporidiosis, are largely unknown (4). For this reason, our reference panel included DNA samples that were positive for six *Cryptosporidium* species, namely, C. hominis, C. parvum, C. canis, C. felis, C. ryanae, and C. scrofarum. All of the species were detected by the VIASURE assay. Additionally, the performance of qPCR tests is largely linked to primer and probe design. Designing diagnostic primers is mainly dependent on intraspecies sequence similarity and interspecies sequence dissimilarity (19). Because intraspecies variation can differ geographically and DNA variation in local subtypes can lead to false-negative test results (15), we devoted special effort to expanding our reference panel with DNA samples belonging to six different *Cryptosporidium gp60* subtype families (Ia, Ib, Ie, IIa, IIc, and IId) and four G. duodenalis assemblages (A, B, C, and F) from clinical samples from different Spanish regions. The VIASURE assay was able to detect and identify all of the aforementioned genetic variants of *Cryptosporidium* and G. duodenalis.

In the present study, the VIASURE *Cryptosporidium*, Giardia, & E. histolytica real-time PCR assay achieved diagnostic sensitivity and specificity for the detection of *Cryptosporidium* spp. of 0.96 and 0.99, respectively. These values were in line with those (1 and 0.99, respectively) estimated in a recent French study with the same multiplex assay and its singleplex version (30). Large differences in the diagnostic performance for the detection of *Cryptosporidium* spp. have been reported for other commercially available multiplex qPCR assays (summarized in Table 1). Whereas sensitivity values of 1 have been achieved with the EasyScreen enteric parasite detection kit (Genetic Signatures, Sydney, Australia) (24) and the FilmArray gastrointestinal panel (BioFire Diagnostics, Salt Lake City, UT, USA) (25), lower values of 0.96 to 0.97 have been reported with the BD MAX enteric parasite panel (Becton, Dickinson and Company) (22, 23), 0.87 with the RIDAGENE parasitic stool panel II (R-Biopharm AG, Germany) (29), 0.74 to 0.75 with the gastroenteritis/parasite panel I (Diagenode, Liège, Belgium) (4, 29, 30), and 0.53 to 0.64 with the FTD stool parasite assay (FAST-Track Diagnostics, Esch-sur-Alzette, Luxembourg) (29, 30). As discussed before, these differences can be attributed, at least partially, to the inability of some assays to detect *Cryptosporidium* species other than C. hominis or C. parvum.

Regarding G. duodenalis, the VIASURE assay achieved diagnostic sensitivity and specificity of 0.94 and 1, respectively. Sensitivity values of 0.81 and 0.96 were previously obtained with the multiplex and singleplex versions of this kit, respectively (30). Other commercially available assays have been demonstrated to be particularly suited for the detection of G. duodenalis in clinical samples; these include the BD MAX (22, 23), FilmArray (25), and NanoCHIP gastrointestinal panel (Savyon Diagnostics, Ashdod, IL, USA) (27, 28) assays, all of which consistently achieve diagnostic sensitivities of >0.97. In contrast, poorer performances (sensitivities of 0.68 to 0.76) were reported for the gastroenteritis/parasite panel I (16, 17).

Finally, the VIASURE assay achieved good diagnostic performance for the detection of E. histolytica, with sensitivity and specificity values of 0.96 and 1, respectively. This is in agreement with previous results obtained using the same assay and its singleplex variant (30). Similar results were also obtained by most commercial kits evaluated to date (Table 1). Slightly lower (0.92 to 0.95) diagnostic sensitivities have been reported using the BD MAX kit (22), the EasyScreen enteric parasite detection kit (Genetic Signatures) (24), and the Luminex gastrointestinal panel (xTAG-GPP; Luminex Molecular Diagnostics, Toronto, Canada) (27) methods. These data should be considered with caution, because the number of E. histolytica-positive samples included in these studies is typically small.

It should be noted that different variables might influence the clinical diagnostic performance of the evaluated commercial assay. In addition to the interspecies and intraspecies genetic diversity discussed above, these factors include panel sample size (small sample numbers are likely to result in inaccurate and inconsistent estimates), amount of parasite DNA available for PCR amplification in the sample (reflecting parasite load and sometimes virulence/pathogenicity), and the diagnostic method used as the gold standard. One of the advantages of this study is the careful selection of a large panel of molecularly (PCR and Sanger sequencing) confirmed DNA samples for testing. Despite this effort, we are aware that some relevant pathogenic and commensal protozoan species were missing from our panel; these include Cryptosporidium meleagridis (the third most common cause of cryptosporidiosis in humans) and potentially cross-reacting species, including Cyclospora cayetanensis, Entamoeba coli, Endolimax nana, and Encephalitozoon intestinalis, among others. Such species should be included in future studies. Quality assessment schemes and multicenter comparative studies are thus necessary to ensure high diagnostic accuracy among the variety of protocols used in different clinical laboratories (15, 20, 23).

Because of superior diagnostic performance (increasing both the positivity rate and the number of coinfections detected) and throughput, reduced turnaround time, and improved laboratory workflows, molecular diagnostic methods are increasingly replacing conventional microscopy as first-line routine diagnostic methods for intestinal protozoan parasites in European clinical laboratories (16, 31, 32). This inexorable trend has some drawbacks that require consideration. Perhaps the most important disadvantage is the inability of PCR-based methods to detect unanticipated cysts, ova, and spores from nontargeted, infrequent pathogenic species such as C. cayetanensis, Isospora belli, and *Encephalitozoon* spp. These species are rarely (≤0.5%) reported in routine diagnosis in European countries, including Belgium (33) and the Netherlands (18). In these scenarios, microscopy may be particularly appropriate and convenient with suspicion of a parasitic infection or in the presence of unresolved or indeterminate results on initial molecular testing.

In conclusion, the VIASURE *Cryptosporidium*, Giardia, & E. histolytica real-time PCR assay (CerTest Biotec) represents a suitable choice for the molecular diagnosis of *Cryptosporidium* spp., G. duodenalis, and E. histolytica during routine clinical practice. Added benefits of this kit include its stabilized, ready-to-use format, reducing the number of time-consuming steps in the laboratory and allowing storage at room temperature.

## MATERIALS AND METHODS

### Ethics statement.

The study design and consent procedures involved in this survey have been approved by the Research Ethics Committee of the Carlos III Health Institute under reference number CEI PI17_2017-v3. All human DNA samples used were anonymized using a unique laboratory identifier code to guarantee the anonymity and confidentiality of the patients. This study was conducted according to the principles set forth by the Declaration of Helsinki and good clinical practice.

### Study design.

This is a comparative, retrospective observational study specifically conducted to evaluate the clinical diagnostic performance of the VIASURE *Cryptosporidium*, Giardia, & E. histolytica real-time PCR assay for the detection and differentiation of *Cryptosporidium* spp., Giardia duodenalis, and Entamoeba histolytica against a large panel (*n* = 358) of well-characterized DNA samples.

### DNA samples.

A panel of DNA samples positive for *Cryptosporidium* spp. (*n* = 96), G. duodenalis (*n* = 115), E. histolytica (*n* = 25), and other parasitic species of the phyla Amoebozoa (*n* = 11), Apicomplexa (*n* = 14), Euglenozoa (*n* = 8), Heterokonta (*n* = 42), Metamonada (*n* = 37), Microsporidia (*n* = 4), and Nematoda (*n* = 6) were included in the study (Table 2). DNA samples were extracted and purified from clinical stool specimens or cultured isolates using the QIAamp DNA stool minikit (Qiagen, Hilden, Germany) during routine testing at the Parasitology Reference and Research Laboratory of the Spanish National Centre for Microbiology (Majadahonda, Madrid) from 2014 to 2019. Human samples were from patients of all age groups (median age, 10.5 years; standard deviation [SD], 14.9 years; range, 1 to 75 years). Some samples were of animal origin, particularly those belonging to animal-adapted species/genotypes or rarely found circulating in humans. All DNA samples were molecularly confirmed by singleplex PCR at initial diagnosis. The singleplex PCR protocols used for the primary detection of *Cryptosporidium* spp., G. duodenalis, and E. histolytica are fully described in the supplemental material. Sanger sequencing was carried out when possible, to identify species and genotypes. All DNA samples were stored at –20°C until testing. The full data set, including all of the information on the DNA samples used and the detailed diagnostic results obtained, can be found in Table S1 in the supplemental material.

### Assay.

The VIASURE *Cryptosporidium*, Giardia, & E. histolytica real-time PCR detection kit (batch VS-KGEXH-021) is designed to amplify a conserved region of the 18S rRNA gene of the investigated pathogens using specific primers and fluorescently labeled probes. The VIASURE *Cryptosporidium*, Giardia, & E. histolytica real-time PCR detection kit contains, in each well, all of the components necessary for the qPCR assay (specific primers and probes, deoxynucleoside triphosphates [dNTPs], buffer, and polymerase) in a stabilized format. The mixture also includes a gene fragment of the enhanced green fluorescent protein (EGFP) as an exogenous internal control (IC) to detect amplification inhibitors and false-negative results in qPCR assays. *Cryptosporidium*, G. duodenalis, E. histolytica, and IC DNA targets are amplified and detected in the Cy5, 6-carboxyfluorescein (FAM), carboxyrhodamine (ROX), and hexachlorofluorescein (HEX) channels, respectively. The assay was performed in strict accordance with the manufacturer's instructions using the DT Prime real-time PCR system (DNA Technologies, Moscow, Russia). Fluorescence was measured at the end of the annealing step of each cycle. The thermal profile used was as follows: step 1, 1 cycle at 95°C for 2 min for polymerase activation; step 2, 45 cycles at 95°C for 10 s and 60°C for 50 s for denaturation and annealing/extension. All DNA samples were blindly analyzed in triplicate to avoid bias. A sample was considered positive if the *C_T_* value obtained was less than 40 and the IC result was positive. Samples yielding *C_T_* values higher than 40 were considered negative even with a positive IC result. A positive control and a negative control provided with the kit were used in each run.

### Analyses.

Cohen’s kappa was estimated to assess the agreement of the diagnostic results obtained with the VIASURE *Cryptosporidium*, Giardia, & E. histolytica real-time PCR detection assay and the reference singleplex PCR methods used during routine analyses at initial diagnosis. Cohen’s kappa ranges between 0 (no agreement between the two raters) and 1 (perfect agreement between the two raters). A Cohen’s kappa value between 0.81 and 0.99 was considered to indicate nearly perfect agreement. Clinical diagnostic sensitivity and specificity and negative and positive predicted values (with 95% confidence intervals) were calculated using MetaDiSc v1.4 freeware software (34) based on the following formulas:

Sensitivity = [a/(a+c)] ×100

Specificity= [d/(b+d)] ×100

Positive predictive value = [a/(a+b)] ×100

Negative predictive value = [d/(c+d)] ×100

where *a* is the number of true-positive samples, *b* is the number of false-positive samples, *c* is the number of false-negative samples, and *d* is the number of true-negative samples. Reference DNA samples that were positive for *Cryptosporidium*, G. duodenalis, and E. histolytica but yielded a negative result in the VIASURE assay were reassessed by singleplex PCR. DNA samples with a negative result in the VIASURE assay but a positive result in the subsequent confirmatory singleplex PCR were considered true false-negative samples.
