# Supplementary material for: Evaluation of a Novel Commercial Real-Time PCR Assay for the Simultaneous Detection of Cryptosporidium spp., Giardia duodenalis, and Entamoeba histolytica
Source: Microbiol Spectr. 2022 May 3;10(3):e00531-22. doi: 10.1128/spectrum.00531-22 (PMC9241732; doi:10.1128/spectrum.00531-22)
Supplement: SUPPLEMENTAL FILE 1 — Supplemental material. Download spectrum.00531-22-s001.pdf, PDF file, 0.04 MB [file spectrum.00531-22-s001.pdf]

ANNEXE I

Singleplex PCR protocols used for the primary detection of *Cryptosporidium* spp., *G. duodenalis*, and *E. histolytica* in clinical samples submitted to the Parasitology Reference and Research Laboratory of the Spanish National Centre for Microbiology, Majadahonda (Madrid).

Molecular detection of *Cryptosporidium* spp.

The presence of *Cryptosporidium* spp. was assessed using a nested-PCR protocol to amplify a 587 bp fragment of the gene codifying the small subunit ribosomal RNA (*ssu* rRNA) of the parasite (1). Amplification reactions (50 µL) included 3 µL of DNA sample and 0.3 µM of the primer pairs CR-P1/CR-P2 in the primary reaction and CR-P3/CPB-DIAGR in the secondary reaction. Both PCR reactions were carried out as follows: one step of 94 °C for 3 min, followed by 35 cycles of 94 °C for 40 s, 50 °C for 40 s and 72 °C for 1 min, concluding with a final extension of 72 °C for 10 min. Sub-typing of the isolates identified as *C. hominis* or *C. parvum* was attempted at the *gp60* gene using the AL-3531/AL-3535 and AL-3532/AL-3534 primer pairs (2).

Molecular detection of *Entamoeba histolytica*

Detection of *E. histolytica* was accomplished by a real-time PCR (qPCR) method targeting a 172 bp fragment of the gene codifying the *ssu* rRNA gene of the parasite (3, 4). Amplification reactions (25 µL) consisted of 3 µL template DNA, 12.5 pmol of the primer set Ehd-239F/Ehd-88R, 5 pmol of each specific TaqMan® probe, and TaqMan® Gene Expression Master Mix (Applied Biosystems, Waltham, MA, USA). Detection of parasitic DNA was performed on a Corbett Rotor GeneTM 6000 real-time PCR system (QIAGEN, Hilden, Germany) using an amplification protocol consisting of an initial hold step of 2 min at 55 °C and 15 min at 95 °C followed by 45

cycles of 15 s at 95 °C and 1 min at 60 °C. We included molecular biology grade water (no-template, negative Nzytech, Lisbon, Portugal) and genomic DNA (positive) controls in each PCR run.

Molecular detection and characterization of *Giardia duodenalis*

Detection of *G. duodenalis* DNA detection was accomplished using a qPCR method targeting a 62 bp region of the gene codifying the *ssu* rRNA of the parasite (5). Amplification reactions (25 µL) consisted of 3 µL of template DNA, 0.5 µM of each primer Gd-80F and Gd-127R, 0.4 µM of probe, and 12.5 µL TaqMan® Gene Expression Master Mix (Applied Biosystems). Cycling conditions and data analysis were as described above for the detection of *E. histolytica*.

Giardia duodenalis isolates that tested positive by qPCR were subsequently re-assessed by sequence-based multi-locus genotyping of the genes encoding for the glutamate dehydrogenase (*gdh*) (6), β-giardin (*bg*) (7), and triose phosphate (*tpi*) (8) proteins of the parasite. We conducted amplifications by semi-nested and nested PCR protocols using the specific primer pairs described in references 6–8.

REFERENCES

1. Tiangtip R, Jongwutiwes S. 2002. Molecular analysis of *Cryptosporidium* species isolated from HIV-infected patients in Thailand. Trop Med Int Health 7:357–364. https://doi.org/10.1046/j.1365-3156.2002.00855.x
2. Feltus DC, Giddings CW, Schneck BL, Monson T, Warshauer D, McEvoy JM. 2006. Evidence supporting zoonotic transmission of *Cryptosporidium* spp. in Wisconsin. J Clin Microbiol 44:4303–4308. https://doi.org/10.1128/JCM.01067-0

3. Gutiérrez-Cisneros MJ, Cogollos R, López-Vélez R, Martín-Rabadán P, Martínez-Ruiz R, Subirats M, Merino FJ, Fuentes I. 2010. Application of real-time PCR for the differentiation of *Entamoeba histolytica* and *E. dispar* in cyst-positive faecal samples from 130 immigrants living in Spain. Ann Trop Med Parasitol 104:145–149. https://doi.org/10.1179/136485910X12607012373759
4. Verweij JJ, Oostvogel F, Brienen EA, Nang-Beifubah A, Ziem J, Polderman AM. 2003. Prevalence of *Entamoeba histolytica* and *Entamoeba dispar* in northern Ghana. Trop Med Int Health 8:1153–1156. https://doi.org/10.1046/j.1360-2276.2003.01145.x
5. Verweij JJ, Schinkel J, Laeijendecker D, van Rooyen MA, van Lieshout L, Polderman AM. 2003. Real-time PCR for the detection of *Giardia lamblia*. Mol Cell Probes 17:223–225. https://doi.org/10.1016/S0890-8508(03)00057-4
6. Read CM, Monis PT, Thompson RC. 2004. Discrimination of all genotypes of *Giardia duodenalis* at the glutamate dehydrogenase locus using PCR-RFLP. Infect Genet Evol 4:125–130. https://doi.org/10.1016/j.meegid.2004.02.001
7. Lalle M, Pozio E, Capelli G, Bruschi F, Crotti D, Cacciò SM. 2005. Genetic heterogeneity at the beta-giardin locus among human and animal isolates of *Giardia duodenalis* and identification of potentially zoonotic subgenotypes. Int J Parasitol 35:207–213. https://doi.org/10.1016/j.ijpara.2004.10.022
8. Sulaiman IM, Fayer R, Bern C, Gilman RH, Trout JM, Schantz PM, Das P, Lal AA, Xiao L. 2003. Triosephosphate isomerase gene characterization and potential zoonotic transmission of *Giardia duodenalis*. Emerg Infect Dis 9:1444–1452. https://doi.org/10.3201/eid0911.030084
